# Recent Trends in the Development of Bone Regenerative Biomaterials

**DOI:** 10.3389/fcell.2021.665813

**Published:** 2021-05-07

**Authors:** Guoke Tang, Zhiqin Liu, Yi Liu, Jiangming Yu, Xing Wang, Zhihong Tan, Xiaojian Ye

**Affiliations:** ^1^Department of Orthopedic Surgery, Tongren Hospital, Shanghai Jiao Tong University School of Medicine, Shanghai, China; ^2^Department of Spine Surgery, The Affiliated Zhuzhou Hospital of Xiangya School of Medical CSU, Hunan, China; ^3^Department of Orthopedic Surgery, Changzheng Hospital, Second Military Medical University, Shanghai, China; ^4^Beijing National Laboratory for Molecular Sciences, Institute of Chemistry, Chinese Academy of Sciences, Beijing, China; ^5^University of Chinese Academy of Sciences, Beijing, China

**Keywords:** regenerative biomaterials, bone defect, tissue engineering, tissue scaffold, 3D printing

## Abstract

The goal of a biomaterial is to support the bone tissue regeneration process at the defect site and eventually degrade *in situ* and get replaced with the newly generated bone tissue. Biomaterials that enhance bone regeneration have a wealth of potential clinical applications from the treatment of non-union fractures to spinal fusion. The use of bone regenerative biomaterials from bioceramics and polymeric components to support bone cell and tissue growth is a longstanding area of interest. Recently, various forms of bone repair materials such as hydrogel, nanofiber scaffolds, and 3D printing composite scaffolds are emerging. Current challenges include the engineering of biomaterials that can match both the mechanical and biological context of bone tissue matrix and support the vascularization of large tissue constructs. Biomaterials with new levels of biofunctionality that attempt to recreate nanoscale topographical, biofactor, and gene delivery cues from the extracellular environment are emerging as interesting candidate bone regenerative biomaterials. This review has been sculptured around a case-by-case basis of current research that is being undertaken in the field of bone regeneration engineering. We will highlight the current progress in the development of physicochemical properties and applications of bone defect repair materials and their perspectives in bone regeneration.

## Introduction

Bone, composed of collagen and calcium phosphate apatite crystals, is the second most commonly transplanted organ worldwide, which provides rigidity, strength, and a certain degree of elasticity to the living body (Turnbull et al., 2018). Various types of bone defects have been developed with major challenges facing the clinical surface, resulting in high demand for bone repair materials (Agarwal and García, 2015). From traditional autogenous bones and allogeneic bones to modern polymer materials and tissue-engineered bones, scientific research and clinical research in related fields have been continuously progressing (Saravanan et al., 2016). However, other than autologous bone with limited bone mass, there are still no ideal materials with simultaneously good biocompatibility, biodegradability, porous three-dimensional structures, bone conduction, osteoinduction, and osteogenesis. According to the development of bone defect repair materials, it can be divided into traditional and modern bone defect repair materials.

Traditional bone defect repair materials mainly include autogenous bone, allogeneic bone, xenogeneic bone, decalcified bone matrix, bioceramics, and metal materials, which are directly sourced with low difficulty in preparation and processing. These bone repair materials possess good biocompatibility, degradability, and a porous three-dimensional structure that benefit from bone conduction, bone induction, and osteogenesis (Enneking et al., 1980; Dick et al., 1985; Stevenson, 1999; Wu and Su, 2000; Boden, 2002). However, in addition to the autogenous bone with limited bone mass, other materials have the disadvantages of immune rejection and low biological activity for the living body. In comparison, modern bone defect repair materials mainly include polymer materials, tissue-engineered bone, and their derived composite materials, which can be designed and fabricated to form the multifunctional bone scaffolds using novel concepts and modern techniques (Langer and Vacanti, 1993; Hutmacher, 2000; Karageorgiou and Kaplan, 2005; Rezwan et al., 2006). With the continuous progress of material sciences and preparation technology, modern materials are associated with seed cells and growth factors that can effectively improve osteogenic ability (Gao et al., 2018; Shi et al., 2019). The popularization of nanotechnology makes materials with a more biomimetic structure through ingenious incorporation of biopolymeric and biodegradable matrix structure with bioactive or easily resorbable nanofillers (Szcześ et al., 2017; Hao et al., 2019; Yin et al., 2019; Singh et al., 2020), and introduction of genetic engineering also significantly stimulates bone repair and regeneration (Malek-Khatabi et al., 2020; Zha et al., 2020). These techniques open up a new prospect for the research of bone defect repair materials. However, modern materials are still in the process of continuous exploration. For example, how to optimize the relevant technology, promote the expression of genes and growth factors, and improve clinical safety and other intractable issues still need to be solved.

Therefore, it is necessary to combine the advantages of different materials and clinical conditions in the selection and application of bone regenerative materials to achieve a better clinical effect and prognosis. Bone regenerative biomaterials are a relatively new class of materials that incorporate a biopolymeric and biodegradable matrix structure with bioactive and easily resorbable fillers that are nano-sized. This review focuses on recent advances in the development and use of traditional and modern bone defect repair biomaterials for bone tissue regeneration.

## Traditional Bone Defect Repair Materials

### Autogenous Bone

Due to excellent bone conduction, osteoinduction, osteogenesis, available source, ideal biocompatibility, and three-dimensional structures, autogenous bone has been regarded as the gold standard in bone defect repair materials. Current clinical treatments to repair bony defects and tissue healing can be problematic due to the anatomy and physiology of bone tissue, as well as the limited knowledge cognition process. Besides the unavailable and limited number of autogenous bones, many major problems are associated with autograft transplantation, such as insufficient tissue, donor-site injury, nerve and vascular injuries, chronic donor site pain, hernias, and surgical risks (bleeding, infection, and chronic pain). Therefore, alternative approaches are urgently needed.

**FIGURE 1:**
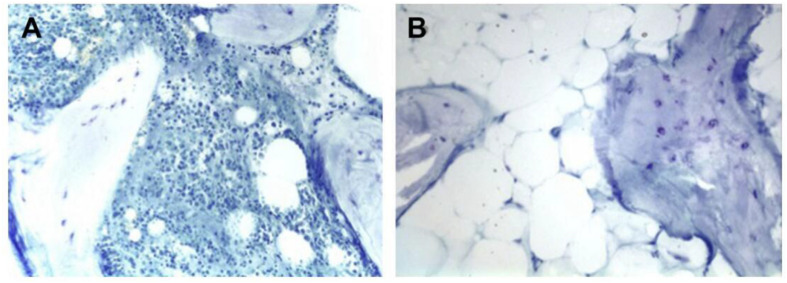
Histologic slides of iliac **(A)** and tibial **(B)** bone graft. The iliac crest bone graft shows abundant osteoblasts and hematopoietic marrow. The tibial bone graft shows fatty marrow without hematopoiesis. Reproduced from Chiodo et al. (2010) with permission from SAGE (Copyright 2010).

The donor site for bone harvesting is the iliac crest, which requires a second surgical intervention and has some surgical morbidity. Although autogenous bone was the gold standard in clinical practice, there are still inevitable disadvantages and potential complications (e.g., insufficient bone mass, size mismatch, low availability, and donor site damage) to limit autogenous bone applications (Mueller et al., 2010). In general, autogenous bone mainly includes cortical bone, cancellous bone, and bone marrow, among which cortical bone is divided into two forms: blood supply and no blood supply. The operation without blood supply is relatively simple, but the lack of blood vessels affects the healing effect (Patwardhan et al., 2013). The operation with blood supply can provide sufficient blood supply and promote bone healing (Niknejad et al., 2008), but the complexity of anatomy makes the operation more difficult (Chatterjea et al., 2010). Transplantation of fresh autologous bone grafts is an attempt to achieve rapid bone repair because living bone can survive well, increase bone volume at the recipient site, and ultimately maintain bone strength. Cancellous bone is capable of bone induction and integration, because it can provide a rich source of bone and marrow cells to promote osteogenesis, and its inherent structural space allows for not only the diffusion of essential nutrients for new bone formation but also limited revascularization through microanastomosis of circulating vessels, but it lacks certain mechanical strength (Hildebrand et al., 1999). In addition, autogenous bone marrow can accelerate vascularization and promote bone repair because it contains mesenchymal stem cells (MSCs) and bone regeneration-related factors (Zhong et al., 2012). Schmitt et al. used calcium phosphate, xenogeneic bone, allogeneic bone, and autogenous bone, respectively, to repair bone defects (Schmitt et al., 2012). After the analysis of imaging and histology, it was found that the osteoinduction and osteogenic ability of these four materials were distinct. Autogenous bone showed the highest osteogenic rate and volume ratio of new bone, which confirmed the strong ability of autogenous bone in bone repair (Miller and Chiodo, 2016). Chiodo et al. carried out a histological study on bone transplantation samples of iliac crest and proximal tibia (Figure 1) and found that iliac crest contained more active hematopoietic marrow than tibia (Chiodo et al., 2010). Similarly, Hyer et al. (2013) found that MSCs in the bone marrow were extracted from the iliac crest, distal tibia, and calcaneus, with a high concentration in the iliac crest. In comparison, autogenous cortical bone graft can be used for the treatment of bone defects that require immediate structural support. Although cortical bone grafts do not reconstruct blood vessels as quickly or properly as cancellous bone grafts, its osteoconduction and surviving osteoblasts in the grafted bone do provide osteogenic properties. Because of these advantages of autogenous bone, it occupies a large proportion in the clinical treatment of bone defect with a success rate of over 90%.

### Allogeneic Bone

Allogeneic bone is an effective substitute of autogenous bone, which has almost no concern about infectious and immune diseases. It can be an alternative of autogenous bone after deep freezing and treatment (Smith et al., 2014). Allogeneic bone has many osteogenic properties; e.g., it prevents the destruction of patients’ healthy bone structure, which can be widely used in bone defects (Wang and Yeung, 2017). However, it still has limitations, such as insufficient supply, ethical disputes, and immunogenicity. Recently, although the bone allografts are taken from a donor in greater quantities with the standard protocols of harvesting, collection, and storage, these grafts integrate more slowly than the autografts. Allogeneic bone with immunogenicity may hinder cell adhesion and differentiation; in addition, its clinical use is relatively limited by complications such as delayed healing and infection (Li et al., 2010). With the continuous advancement of preparation technology, allogeneic bone is currently widely used in the form of freeze-dried allogeneic bone. Wei et al. (2013) used two kinds of freeze-dried allogeneic bone to treat the femoral bone defect in rats. Through micro-CT scanning and histological analysis of 8 weeks after operation, it was found that both of them continued to form new bone, verifying their good osteoinduction and osteogenic ability. In recent years, many researchers have also confirmed that decalcified freeze-dried allogeneic bone has growth factors to promote bone induction, which can aggregate MSCs and support osteoblast differentiation (Clark et al., 2019; Tresguerres et al., 2019). Thus, it has been the only biomaterial approved by the Food and Drug Administration (FDA) in North America for clinical bone repair (Soardi et al., 2012). Although these recent strategies like sterilization and freezing can effectively minimize the potential risk of infectious agent transmission of the allogeneic bones, these procedures in turn reduce graft performance in terms of osteoinduction, bone conduction, and mechanics (Shang et al., 2021).

### Xenogeneic Bone

Xenogeneic bone is widely derived, but due to the antigens of different species, it must be treated artificially to avoid possible immune rejection after transplantation (Ghanaati et al., 2014). In addition, the potential pathogenicity and related ethical issues have yet to be resolved; for example, these heterologous grafts may put patients at risk of immune rejection and virus transmission. The treated xenogeneic bone can retain some biomechanical properties for bone repair, but lose the ability to induce the differentiation and proliferation of MSCs. Recently, Kubosch et al. (2016) analyzed 232 patients with bone defects over a 10-year period, including 116 allogeneic cancellous bone and 116 synthetic allogeneic bone. The results showed that both materials could promote bone healing, but the osteogenic ability of xenogeneic bone was relatively poor, reflecting the lack of biological activity and osteoinductive ability of heterogeneous bone. Therefore, researchers have proposed a method to combine other repair materials or related factors, including complex bone morphogenetic protein, autologous bone marrow, and growth factors, which have become a hot spot in the research of heterogeneous bone. On the premise of avoiding immune rejection, it strengthens the ability of bone induction and promotes bone healing (Del Deo et al., 2018; Li et al., 2018). With the development of related research and technology, compound xenotransplantation will receive more and more attention. Theoretically, the availability of xenogeneic bones is unlimited despite the possibility of zoonosis transmission if they can be handled for the host (Oryan et al., 2014). These two allografts and xenografts have been decellularized to reduce antigenicity, leading them to fall under the category of tissue engineering. It was mentioned that other factors like the implanted site location or epidemiological parameters also influenced the osteogenic ability. Therefore, the combination of other repair bioactive sources, xenogeneic materials or complex morphogenetic protein, autologous bone marrow, and other factors may be an attractive strategy to promote bone regeneration.

### Demineralized Bone Matrix

**FIGURE 2:**
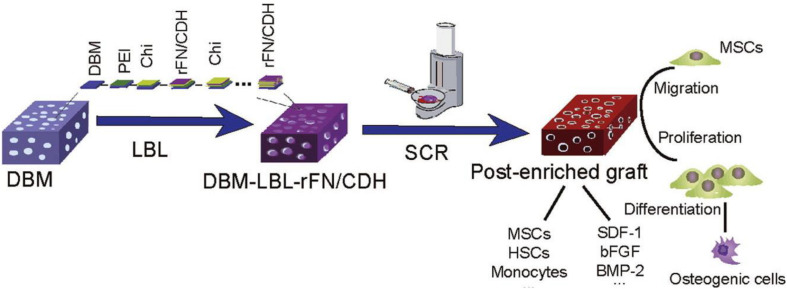
Modified demineralized bone matrix with nanoscaled and multi-layered recombinant fibronectin/cadherin chimera for bone repair. Reproduced from Xing et al. (2017) with permission from Elsevier (Copyright 2017).

The demineralized bone matrix for clinical application is mainly derived from donor allogeneic bone, including collagen (mainly type I and type IV), non-collagen, growth factors, a small amount of calcium phosphate, and cell debris (Gruskin et al., 2012). The demineralized bone matrix is used as a bone repair matrix and a carrier for delivering bioactive agents. Bone morphogenetic protein is exposed and released after decalcification and artificial treatment, which can induce bone formation in the decalcified bone matrix and promote bone regeneration (Schubert et al., 2013). However, the osteoinduction of demineralized bone matrix is negatively correlated with its antigenicity. Excessive reduction of antigen in artificial treatment can destroy many osteogenic factors and significantly reduce the osteogenic properties. In addition, due to the loss of a large number of inorganic components and the corresponding biomechanical properties, the demineralized bone matrix is not suitable for the repair of bone defects in load-bearing areas (Burg et al., 2000). The demineralized bone matrix also has good bone conduction ability, histocompatibility, and pore structure. Among them, appropriate biological pore structure can benefit the slow release of bone morphogenetic protein, facilitate the attachment and growth of osteoblasts and factors, and promote bone regeneration (Xie et al., 2017). Controlling the pore size is a problem that needs to be addressed, because the pore size of demineralized bone matrix (approximately 200–500 μm) is larger than the size of cells, which was on the order of tens of micrometers. Hou et al. (2014) combined the demineralized bone matrix with nano self-assembled peptides to reduce the pore size, enhance the charge interaction, and increase the number of osteoblasts and factors to enrich the material with osteogenic stem cells and growth factors. At the same time, the materials can also establish the microenvironment of cell adhesion, proliferation, and differentiation, thus improving the osteogenic ability (Hou et al., 2014). Recently, due to the rise of composite bone repair materials, demineralized bone matrix has rapidly become one of the most mainstream scaffold materials (Van Bergen et al., 2013). Xing et al. (2017) innovatively used layer-by-layer (LBL) self-assembly technology to modify nano-layered recombinant fibronectin/cadherin chimera to the demineralized bone matrix (Figure 2). It was found that the composite material significantly improved the efficiency of cell selection and retention through the physical interception and chemical recognition, which provided a favorable microenvironment to promote the migration, proliferation, and osteogenic differentiation of MSCs. In addition, these biomaterials were cost-effective, were easy to store and transport, and could be constructed quickly during the surgery (Xing et al., 2017). Munir et al. (2019) prepared a functional poly(L-lactide-co-epsilon-caprolactone) scaffold by 10 or 20 μg/ml of human demineralized dentine matrix. After culture of 7 and 21 days on human bone marrow stromal cells in basal medium or non-functionalized scaffolds in osteogenic medium, the human bone marrow stromal cells proliferated less in demineralized dentine matrix and activated ERK/1/2, exhibiting highest expression of IL-6 and IL-8 at 7 days and higher collagen and bone morphogenetic protein-2 at 21 days, indicating the signs of mineralization that provided a promising approach on stimulating osteogenic differentiation of human bone marrow stromal cells. Wang et al. (2019) reported a robust silicification strategy on fabrication of an osteoinductive and porous collagen scaffold via a GF-free and one-step surgery for *in situ* bone regeneration. This composite scaffold possessed a native-bone-like porous structure and a nano-silica coating. Without usage of any exogenous cells and growth factors, this decellularized scaffold benefited from its surface roughness (topographic signal) and silicon content (chemical cue) and synergistically activated multiple signaling pathways related to MSC recruitment and bone regeneration, which enabled large-size, complex porous, and varied osteoinductivity, exhibiting great potentials for clinical translation in massive bone repair.

### Bioceramics

Bioceramics are a kind of inorganic non-metallic materials. Because the main components of bone tissue are hydroxyapatite (HAp), the typical biomaterials of calcium phosphate ceramic (CaP) and bioactive glasses (BG) are widely used as bone substitutes for many years. Their mimicry of the mineral phase endows the bone with bioactivity for new tissue formation (Kokubo et al., 2003; El-Ghannam, 2005).

#### Calcium Phosphate

**FIGURE 3:**
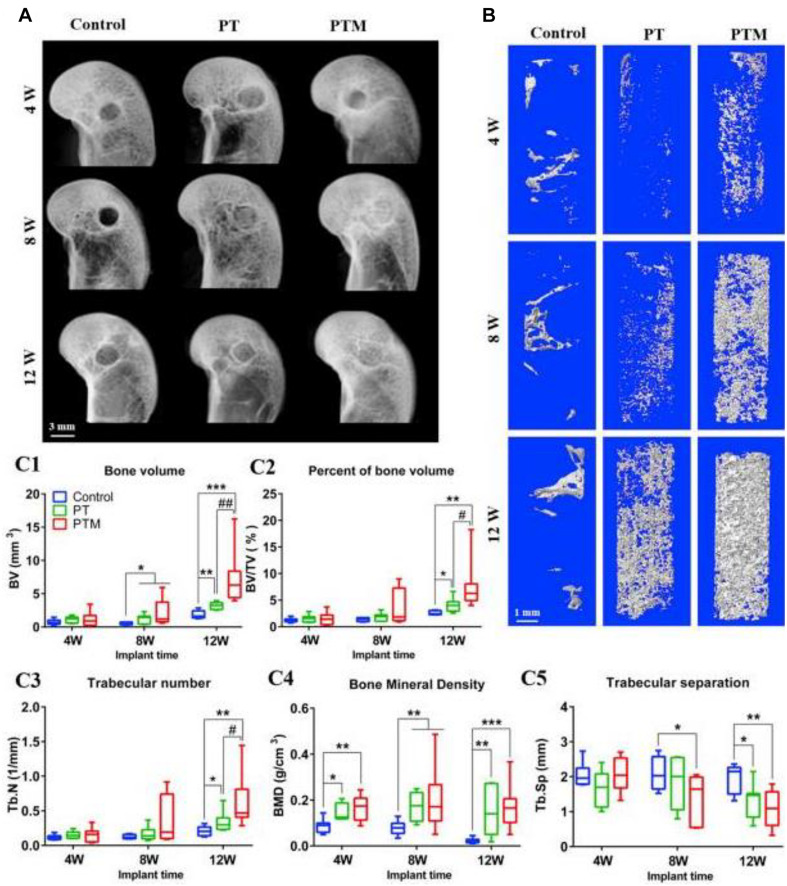
New bone formation within bone tunnel. **(A)** Representative radiographs at 4, 8, and 12 weeks after surgery. **(B)** Representative 3D micro-CT images within a region of interest of central 2.5 mm in diameter of the bone tunnel at 4, 8, and 12 weeks after surgery. **(C)** Quantitative analysis of micro-CT of the new bone in the bone tunnel at 4, 8, and 12 weeks after surgery: **(C1)** BV; **(C2)** BV/TV; **(C3)** Tb.N; **(C4)** BMD; **(C5)** Tb. Sp. *n* = 8. *p < 0.05 vs. control group, **p < 0.01 vs. control group, ***p < 0.001 vs. control group, ^#^*p* < 0.05 vs. PT group, ^##^*p* < 0.01 vs. PT group. Reproduced from Lai et al. (2019) with permission from Elsevier (Copyright 2019).

Calcium phosphate has various forms of ceramic, powder, and bone cement, mainly including α-tricalcium phosphate, β-tricalcium phosphate (β-TCP), tetracalcium phosphate, etc., among which β-TCP is a most commonly used biomaterial. Although calcium phosphate has excellent prospects on good bone conductivity, resorption, and biocompatibility for promoting bone repair (Pina et al., 2015; Fukuda et al., 2017; Xu et al., 2017), there are many shortcomings in calcium phosphate itself. For example, the limited mechanical strength and high brittleness are still the most prominent shortcomings of the material, so it can only be used in the non-weighted area (Castro et al., 2017). Besides, a suitable degradation rate and an appropriate curing time prove to be difficult for further research and breakthrough of calcium phosphate. It is worth mentioning that various forms of calcium phosphate have significant advantages in flexibly coping with various types of bone defects. Lai et al. (2019) used low-temperature rapid prototyping (LT-RP) technology to make a new porous PLGA/TCP/Mg (PTM) scaffold, which had the appropriate physical structures and mechanical properties to meet the initial needs of bone regeneration and tissue repair. When MG was combined with PT, PTM scaffolds not only provided an appropriate template for vascular crawling but also promoted angiogenesis, ultimately mediating new bone formation and remodeling while challenging the association between bone defects and steroid-related osteonecrosis (Figure 3).

In addition, researchers have developed a new injectable form of calcium phosphate cement, composed of the calcium phosphate solid phase and blood and other liquid phases. After precipitating reaction and crystal entanglement, calcium phosphate cement can be used to solidify the defect. Therefore, the injectable form of calcium phosphate bone cement for matching with various defects has attracted extensive attention (Zhang et al., 2014, 2016; Yan et al., 2019; Raucci et al., 2020). Luo et al. (2018) developed an injectable ready-to-use two-phase system consisting of a monocalcium phosphate monohydrate paste and a β-TCP paste based on acidic cement. Because of good cohesion, compressive strength, and adequate shelf life, it showed great potential in a dual-chamber system for simplified and fast filling of bone defects in a minimally invasive manner, which significantly reduced surgery time, decreased the risk of contamination, and ensured repeatable results.

**FIGURE 4:**
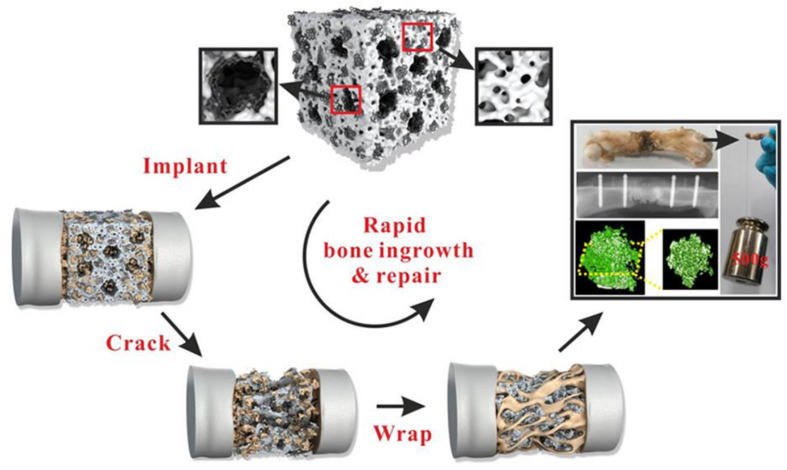
Diagram of the formation mechanism of porous HA/rGO composite scaffold. Reproduced from Zhou et al. (2019) with permission from the American Chemical Society (Copyright 2019).

HAp, a calcium phosphate bioceramic, is an essential component for bone regeneration possessing good biocompatibility, bioactivity, and bone conductivity that has been widely used in biomedicine and bone defect repair materials (Szcześ et al., 2017; Farokhi et al., 2018; Mao et al., 2018). With the rapid development of nanomaterials technology, HAp with a nanoscale size, termed nano-HAp (n-HAp), can obtain high surface activity and ultrastructure (Wei and Ma, 2004; Wu et al., 2015), which has higher absorbability and biological activity to favor the cellular response compared with traditional HAp (Atak et al., 2017). Therefore, n-HAp has the ability to exhibit advanced performance in proliferation and differentiation of osteogenic-related cells for bone regeneration (Nie et al., 2017). To further improve the properties and activities of n-HAp, researchers have tried to integrate n-HAp with other biomaterials using advanced technologies (Venkatesan and Kim, 2014; Jakus et al., 2016; Ao et al., 2017; Zhu et al., 2017; Lu et al., 2018). For example, Zhou et al. (2019) prepared hierarchical porous HA/rGO scaffolds by combining the reduced graphene oxide (rGO) with HA through a soft template method (Figure 4). The scaffold had a graded pore structure, a nano surface, a suitable porosity and pore size, and good biomechanical properties. The graded pore structure was conducive to cell adhesion, fluid exchange, and cell inward growth. rGO could improve cell adhesion and promote cell proliferation and osteogenic differentiation of bone marrow mesenchymal stem cells (BMSCs). The degradation rate of HA/rGO composite scaffolds was well matched with the rate of new bone formation. Therefore, porous HA/rGO composites were a kind of excellent bone defect repair scaffold for tissue engineering (Zhou et al., 2019).

Li et al. (2019) reported a well-organized, terbium (Tb)-doped HAp nanocrystal for bone repair. By tracing the changes of morphology, composition, and structure of implanted HAp-Tb in the process of bone reconstruction, the detailed changes *in vivo* were revealed, which were not achieved by the conventional irregular HAp particles or nanocrystals. Adding a certain amount of Tb ions to HAp provided element tracking, micro-CT imaging, and fluorescence imaging capabilities, which could not change the crystal structure, morphology, and biocompatibility of HAp. These results help us to understand the characteristics of HAp crystal and bone apatite crystal, and guide the design of new bionic bone repair materials. The development of smaller sub-n-HAp crystals will be an important research direction in the future, which has important scientific value and clinical significance for the development of biological characteristics and applications of HAp biomaterials.

#### Bioactive Glass

In addition to calcium phosphate, bioactive glass with main components of Na_2_O, CaO, SiO_2_, and P_2_O_5_ is also widely used for load-bearing bone repair because of its high bioactivity, bone binding ability, and mechanical properties (Hench, 2006). When the bioactive glass materials made contact with body fluid, they can generate an HA-like layer to form a stronger interface between the material scaffolds with surrounding hard and soft host bone tissues (Lee et al., 2011). To reduce its brittleness, bioactive glass can be fabricated with suitable pore structures by optimizing composition, processing, and sintering conditions to well-match the human trabecular bone and cortical bone. In addition, introduction of metallic ions (e.g., Cu, Co, Si, Zn, and Mg) could improve the mechanical properties and enhance bioactivity. For example, the addition of Cu to a mesoporous bioactive glass scaffold can effectively induce angiogenesis and promoted MSC osteogenesis (Wu et al., 2013). Quinlan et al. (2015) demonstrated that the introduction of cobalt could also improve angiogenesis and osteogenesis. As an essential element for the mineralization of osteoblasts, silicon-based bioceramics expressed outstanding effects on bone regeneration (Li et al., 2017). Song et al. (2020) reported that the addition of zinc silicate to composites of collagen and HAp could improve bone angiogenesis, manipulate the monocytes, and generate the osteogenic microenvironment.

### Metallic Materials

Metallic materials are mainly used for parts requiring mechanical support, such as long bone (femur, tibia, etc.) and bone defects of vertebrae. These metal materials need to be tightly bound to bone to provide a physiological load on the implant site for wide application. The main problem is that the corrosion of physiological environment can change the properties of materials and improve the level of metal ions *in vivo*, leading to implant failure and potential side effects. Therefore, an ideal metal material should have excellent biocompatibility, safety, and corrosion resistance (Navarro et al., 2008; Zheng et al., 2014; Chen and Thouas, 2015). As a typical representative, titanium, magnesium, tantalum, and their alloys are more mature in clinical application.

#### Inert Metals

Titanium (Ti) and Ti-based alloys are widely used in orthopedic implants because of its bone tissue-like structure, high mechanical performance, and excellent biocompatibility (Albrektsson et al., 1986). However, there was lack of sufficient osseointegration originating from the unsatisfied bioactivity, corrosion resistance, and mechanical mismatch problems with bone tissues. The inertness of Ti could easily cause the formation of fibrous tissue and raise the loosening risk during the long-term usage while the poor corrosion resistance led to the dissolution of Ti into the body to hinder bone healing and intensify the release of inflammatory cytokines, thus resulting in chronic inflammation and implant loosening. The mechanical mismatch cannot provide proper mechanical stimulus for bone lining cells of osteoblastic origin and osteocytes, which cannot produce enough biochemical signals to conduct the acquired mechanical signals and regulate bone formation and absorption. So, it is necessary to enhance its biological activity, corrosion resistance and mechanical mismatch to enhance osseointegration through surface coating, including biological adhesive coating and composite coating, which are the basic and indispensable demands in clinic applications (Thukkaram et al., 2020; Xing et al., 2020).

**FIGURE 5:**
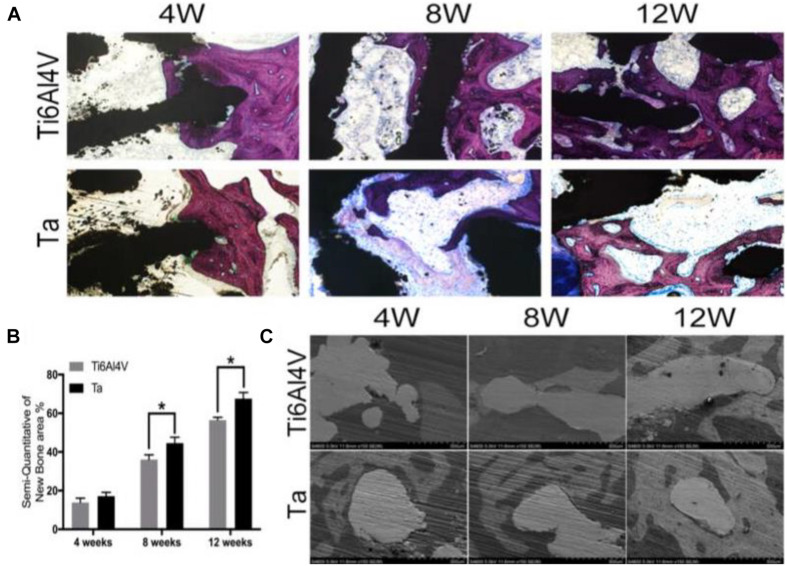
Hard tissue section stained by van Gieson staining **(A)** and histomorphometric analysis **(B)** of Ti6Al4V and Ta scaffolds at 4, 8, and 12 weeks after surgery. The red-stained tissue represents bone tissue; at 4 weeks, the amount of new bone tissue in the scaffolds is thin and irregular. Osteoblasts seam with bone-lining cells, indicating active bone formation. *P < 0.05, vs. Ti6Al4V group. **(C)** SEM images of bone apposition and bone microstructure on porous scaffolds at di?erent positions at 4, 6, and 12 weeks. White: implant; gray: new bone. Reproduced from Guo et al. (2019) with permission from the American Chemical Society (Copyright 2019).

In recent years, a new type of “bone trabecular metal”-porous tantalum (Ta) has attracted great attention, because it has good biocompatibility, ideal modulus of elasticity, corrosion resistance, and high porosity (Han et al., 2019), which promote cell adhesion, growth, and differentiation; form rich extracellular matrix; and enhance the early biological fixation in both research and clinical applications (Balla et al., 2010a, b; Fraser et al., 2019; Tang et al., 2020). Guo et al. (2019) used selective laser melting (SLM) technology to manufacture porous Ta scaffolds with a pore size of 400 μm. The porous Ta scaffold was implanted into a cylindrical bone defect with a height and diameter of 1 and 0.5 cm, respectively, in the lateral femoral condyle of New Zealand rabbits. Radiographic analysis showed that the new bone formation in Ta scaffolds was higher than that in Ti6Al4V scaffolds (Figure 5). The porous Ta scaffold manufactured by SLM not only had a regular pore shape and connectivity but also had controllable elastic modulus and compressive strength. Moreover, *in vitro* and *in vivo* osteogenesis and osseointegration results were improved compared with those porous Ti6Al4V scaffold manufactured using the same technology. Therefore, tantalum-related products have been applied in the field of orthopedics and achieved encouraging results, which is expected to be developed as an excellent bone defect repair material. Although porous Ta is important in orthopedic application via various manufacture technologies, the anatomical shape and microstructure can only be designed and fabricated in a limited scope. Furthermore, porous Ta implant customization was difficult to realize due to cost and efficiency.

#### Biodegradable Metals

As a kind of metal with good biocompatibility, biodegradation, and osteogenesis, magnesium (Mg) and its alloy implants have a broad application prospect in fracture fixation (Sedghi et al., 2019; Putra et al., 2020). Mg is recognized as a degradable metal with similar Young’s modulus to the cortical bone for facilitating bone regeneration. Huang et al. (2020) used high-purity Mg screws to fix and study the fracture of femoral neck in goats. The results showed that high-purity Mg screw had good mechanical strength, degradation, and osteogenesis, which provided a basis for the clinical transformation of high-purity Mg-bearing screws. In addition, iron (Fe)-based alloys can be used as bone regenerative implants. Chou et al. (2013) prepared Fe-Mn composite scaffolds via inkjet 3D printing technology, which had similar mechanical properties and biodegradability to the cancellous bone, thus allowing cells to penetrate the porous structure.

In fact, all the implanted porous structures were fabricated with homogeneous and regular microstructures, but the gradient pore structure design (size, porosity, and randomization) should also be applied for better biomechanics and biocompatibility on bones. Gradient and controllable design of metallic materials with both anatomically macroscopic anatomical shape and microscopic bionic structure might be the focus in the next few years. Besides, infection still seemed to be a perennial theme in orthopedics for implants. To make porous metal implants with better mechanics, cell proliferation, and antibacterial and antitumor properties, it is necessary to continue to modify the surface of porous metal implants in the future.

## Modern Bone Defect Repair Materials

### Polymer Materials

#### Natural Polymers

The field of bone tissue engineering (BTE) is a paradigm that aims to successfully incorporate regeneration of bone at defect sites of the host without any additional complications, such as donor site morbidity, immunogenicity, and poor vascularization. BTE employs biocompatible and biodegradable natural materials to provide suitable bioactive environments and necessary mechanical support to promote the growth of new bone tissue in defect sites. Due to their superior biocompatibility and minute negative immunological influence, natural polymers such as chitosan, collagen, gelatin, hyaluronic acid, alginate, and fibroin are extensively used in BTE research. However, they have insufficient mechanical strength, rapid degradation rate, unstable biological properties, and limited production capacity, and therefore, these materials are difficult to design, process, and apply for bone defect repair (Venkatesan et al., 2015; Jahan and Tabrizian, 2016; Melke et al., 2016; Saravanan et al., 2018; Kashirina et al., 2019; Ranganathan et al., 2019; Zhai et al., 2019; Kołodziejska et al., 2020). As a representative, silk fibroin (SF) has shown a good prospect in BTE due to its excellent biocompatibility, high porosity, and good mechanical properties (Mottaghitalab et al., 2015; Saleem et al., 2020). In addition, the degradation speed *in vivo* matches the repair cycle of bone defect, showing great advantages in bone defect material (Farokhi et al., 2018; Kwon and Seok, 2018). Therefore, the scaffold material based on SF has been widely studied by researchers worldwide.

**FIGURE 6:**
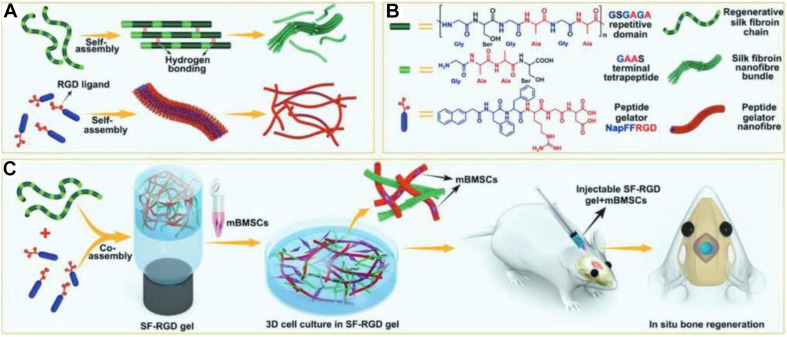
**(A,B)** Molecular structures and self-assembling properties of peptide gelator and SF for the formation of nanofiber and nanofibril bundle structures. **(C)** Schematic of preparation process of SF-RGD for bone regeneration in calvarial defect areas of mouse. Reproduced from Yan et al. (2018) with permission from Wiley (Copyright 2018).

Perrone et al. (2014) prepared a new type of absorbable SF scaffold with good biocompatibility for tissue repair. The absorbability of material avoided the shortcomings of secondary removal and stress shielding and further improved the ability of bone repair (Perrone et al., 2014). Yan et al. (2018) had developed a functional silk fibroin hydrogel (SF-RGD) using small molecular peptides (NapFFRGD) as gelling agents (Figure 6). On account of the presence of many RGD in SF-RGD hydrogels, these biocompatible hydrogels not only promoted the osteogenic adhesion and differentiation of mesenchymal stem cells but also provided a bionic microenvironment for bone regeneration using the mouse skull defect models (Yan et al., 2018). Bai et al. (2019) proposed a designable strategy to construct a new type of bone cement that could provide stable fracture fixation and accelerate bone regeneration during bone remodeling. The adhesive was used as a phenolic resin with tannic acid (TA) and spontaneously co-assembled with SF and HAp to obtain inorganic–organic hybrid hydrogel (SF@TA@HAp). This adhesive not only fixed the bone fracture *in vivo* with timely mechanical repair but also accelerated bone regeneration. Therefore, with the development of SF research, the SF composite hydrogel was expected to become an ideal scaffold for generation of bone defect repair materials. Scientists have learned that the human body is a tremendous potential source of biomaterials for effective therapeutics, and these natural polymers have made great progress in bone regenerative medicine. In recent years, non-collagen proteins from bone ECM were combined with 3D nanofibrous gelatin scaffolds to form a material device that could mimic the chemical composition and nanostructural architecture of the natural bone ECM. Sun et al. (2013) reported that the introduction of these non-collagen proteins could effectively improve the osteogenesis and mineralization for new bone regeneration. El-Fiqi et al. (2020) reported a bone-mimetic nanohydroxyapatite/collagen porous hybrid scaffold. The presence of nanobioglass in the fibrillar collagen network promoted the growth of HA crystals and maintained the porosity of collagen scaffold, which demonstrated that the mineralized scaffold had a favorable osteogenic potential for the calvaria bone defect repair (El-Fiqi et al., 2020).

#### Synthetic Polymers

**FIGURE 7:**
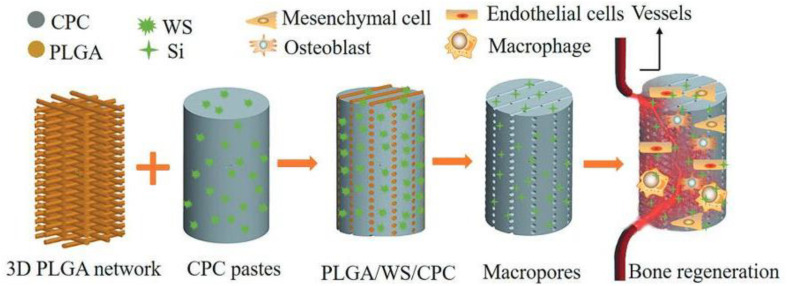
Schematic diagram of calcium phosphate-based composite cement with an embedded 3D plotted PLGA network and bioactive wollastonite for osteogenesis and angiogenesis. Reproduced from Qian et al. (2019) with permission from Wiley (Copyright 2019).

Specific applications in bone tissue regeneration require certain modifications to the polymer structure. Compared with natural polymers, synthetic polymers have poor biocompatibility, weak hydrophilicity, and cell adhesion, and may cause aseptic inflammatory responses. However, the mechanical properties of synthetic polymers are relatively better than those of natural polymers, and their fixed component proportion and processing properties are also advantageous. As star synthetic polymers, polylactic acid (PLA), polycaprolactone (PCL), and poly(lactic-co-glycolic acid) (PLGA) are widely used in the form of scaffold materials, but their degradation products may cause the proliferation of degradation rate and the occurrence of inflammatory reaction (Athanasiou, 1996; Woodruff and Hutmacher, 2010; Danhier et al., 2012). For example, hydrolytic degradation of PLA is attributed to the breakdown of ester bonds within molecular chains under the action of hydrogen ions to form alcohols and carboxylic acids. The generated acid has a catalytic effect on degradation with an autocatalytic effect, and these carboxylic acids cause a local acidic microenvironment that is harmful to cell proliferation and bone repair, which can produce a potentially inflammatory reaction. In addition, when such materials are used as bone defect repair materials, it is necessary to composite growth factors, cells, or other materials to improve biological activity to facilitate cell adhesion and proliferation (Yassin et al., 2017; Barati et al., 2020; Bharadwaz and Jayasuriya, 2020). The composite scaffolds composed of degradable synthetic polymers and bioceramics have aroused great interest in many researchers. Biodegradable polymer materials have tough structures while bioceramics improve the electrical conductivity of bone, thus allowing for flexible adjustment of its composition and microstructure while maintaining its respective advantages (Puppi et al., 2012; Dos Santos et al., 2019; Alksne et al., 2020; Zhu et al., 2020). Qian et al. (2019) infiltrated pastes containing calcium phosphate bone cement (CPC) and wollastonite (WS) into a 3D plotted PLGA network to fabricate plastic CPC-based composite cement (PLGA/WS/CPC) for the first time. The PLGA/WS/CPC recovered the plasticity of CPC after being heated above the glass transition temperature of PLGA (Figure 7). The presence of PLGA network significantly increased the flexibility of CPC in prophase and generated 3D interconnected macropores *in situ* upon its degradation. The addition of WS was helpful to improve the attachment, proliferation, and osteogenic differentiation of mouse bone marrow stromal cells *in vitro*. The *in vivo* results indicated that PLGA/WS/CPC could promote rapid angiogenesis and bone formation with good mechanical properties and cell com6patibility, which provided a new direction for the development of scaffold of bone defect repair materials.

### Composite Materials

Composite materials are a combination of two or more materials with different morphology or composition at the micro-/nanoscale. Due to the limitations of a single material in biological, physical, and chemical properties, composite biomaterials have combined advantages on improving biological characteristics and multiple performances for bone regeneration. Composite materials are mainly divided into composite of various materials (such as composite between bioceramics and polymer materials), composite of preparation technology and materials, and composite of tissue engineering technology and materials (Chen et al., 2019; Zhang et al., 2020). We use collagen as an example to demonstrate the importance of composite materials on bone regeneration. Collagen consisting of several triple-helical chains has been widely used as BTE scaffolds because of the excellent biocompatibility, easy bone formation, and remodeling process, but it generally possesses low mechanics and osteoinductivity. To address this issue, many various agents were incorporated to largely improve the porosity, stability, osteoinductivity, and osteogenicity of composite matrixes in bone regeneration. In general, there are five therapeutic targets in bone regeneration, such as vascularization, growth factors, osteogenesis, osteoconductive scaffolds, and mechanical environment (Zhang et al., 2018). Wu et al. (2020) prepared a biomimetic and osteogenic composite scaffold (3DS) with HAp and nano magnesium oxide (MgO) embedded in fiber (F) of silkworm cocoon and SF for bone regeneration. On account of the combined effect of HAP and MgO, magnesium ions (Mg^2+^) promoted bone mesenchymal stem cell (BMSC) proliferation, osteogenic differentiation, and alkaline phosphatase (ALP) activities while HAp provided outstanding osteoconductive properties, which were used as potential 3D composite scaffolds for bone regeneration applications (Wu et al., 2020).

Additionally, unique hierarchical structures of biological composites had been applied for design of the high-performance materials with excellent mechanical properties. The combination of organic polymers and inorganic minerals was a promising approach to improving mechanical performance. For example, composite materials from chitosan and inorganic minerals have been applied as porous scaffolds especially in bone regeneration. Chitosan/calcium phosphate composites can achieve high interaction between the bioactive calcium phosphate phase and the chitosan to obtain a tough material for BTE (Salama et al., 2016). Various techniques have been investigated to synthesize calcium phosphate materials such as freeze casting, vacuum-assisted filtration, and biomimetic mineralization (Jafarkhani et al., 2012; Sumathra et al., 2018). For example, a double diffusion method was applied to assist the growth of HAp crystals onto three-dimensional porous chitosan scaffolds. In situ hybridization by ionic diffusion processes was investigated for preparing transparent chitosan/HAp nanocomposite for internal fixation of bone fracture (Hu et al., 2004; Manjubala et al., 2006). Kaneko et al. (2020) prepared a composite material scaffold based on the octacalcium phosphate/weakly denatured collagen for improving the osteo-regenerative effect in a canine model. Octacalcium phosphate was prepared using Ca-acetate and NaH_2_PO_4_, and the octacalcium phosphate particles with a diameter of 199–298 μm were mixed with a collagen matrix to create an octacalcium phosphate/weakly denatured collagen scaffold. After implanting this octacalcium phosphate/weakly denatured collagen into the defects, bone regeneration was evaluated via histopathological analysis, which revealed the osteoblast infiltration and osteo-regeneration in all defects for bone reconstruction.

**FIGURE 8:**
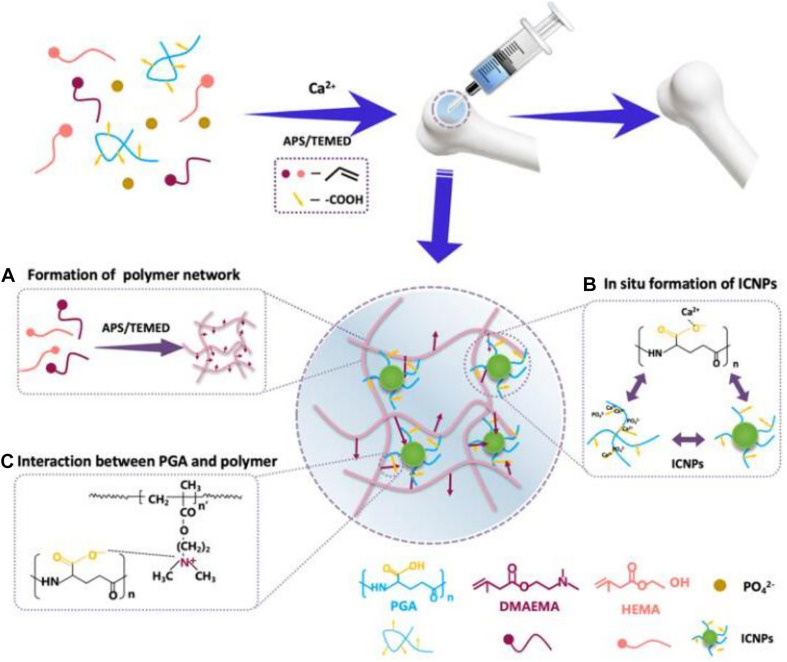
**(A)** Formation of the polymer network from HEMA and DMAEDA via Michael addition reaction (purple arrows: positive charges of DMAEMA). **(B)** *In situ* self-assembly of CaP NPs around -COOH group of PGA via -COO^–^ -Ca^2+^ coordination. **(C)** The interaction between the PGA and the polymer network via electrostatic attraction. Reproduced from ref. Kuang et al. (2019) with permission from the American Chemical Society (Copyright 2019).

The application of modern preparation technology in bone defect materials has been developing rapidly. Wu et al. (2019) constructed a composite periosteum with slow-release vascular endothelial growth factor (VEGF) by combining electrospinning with collagen self-assembly. This biomimetic periosteum could be used alone or combined with the existing bone grafting materials to reduce the phenomenon of non-union in clinical bone defects. As a guided tissue regeneration (GTR) membrane, this biomimetic structure had great clinical and commercial value. Kuang et al. (2019) prepared an injectable nanocomposite hydrogel by injecting two methylamino ethyl methacrylate (DMAEMA) and 2-hydroxyethyl methacrylate (HEMA) into polymer PDH during *in situ* growth of calcium phosphate nanoparticles (ICPN) (Figure 8). The self-assembly of ICPN was achieved by adding poly(L-glutamate) (PGA) that can bind calcium ions as nucleation sites to form calcium phosphate nanoparticles. In addition, BMSC-specific aptamers (APT19s) were covalently anchored to hydrogels to enhance the material’s ability to capture BMSCs (Kuang et al., 2019). The integration of these new technologies and materials had opened a new direction for developing the next generation of bone repair materials.

### Tissue-Engineered Bone

Tissue-engineered bone is mainly composed of four key components: (1) osteoblasts that can give rise to the matrix of bone tissue; (2) a biocompatible framework or scaffold from a bioactive material that can mimic ECM; (3) vascularization that can offer mass transport of nutrients and wastes; and (4) capacity that can guide cell morphogenesis signals.

#### Seed Cells and Growth Factors

Seed cells are the main source of biological activity in tissue-engineered bone. Ideal seed cells need a wide range of sources, low preparation difficulty, and excellent osteogenic potential and proliferation ability, which can differentiate into osteoblasts and flexibly adapt to various microenvironments (Yousefi et al., 2016). The most widely used seed cells are MSCs from bone marrow, adipose tissue, and peripheral blood. BMSCs remain the preferred source of materials with strong proliferative ability to differentiate into osteoblasts and chondrocytes, thereby avoiding immune rejection and pathogenic defects by other cells (Pittenger et al., 1999; Jiang et al., 2002; Lee et al., 2004).

Growth factor can regulate the proliferation and differentiation of cells as well as the synthesis of extracellular matrix. This effect is mainly through the early autocrine and paracrine way to improve proliferation rate and activity of the osteoblasts, significantly enhancing regeneration ability (Marx et al., 1998; Street et al., 2002). The common growth factors include insulin-like growth factor, platelet-derived growth factor, and bone morphogenetic protein. Among them, bone morphogenetic protein is firstly isolated and found by Urist as a most widely applied factor (Urist et al., 1983). It is proved that BMP2 and BMP7 are effective in the treatment of bone defects (Sun et al., 2012). However, how to improve and optimize the binding efficiency of seed cells and scaffolds and the corresponding clinical effects still need to be further verified and resolved in the future.

#### Scaffold Materials and Nanomaterials

Scaffold materials are an important center for the tissue-engineered bone. An ideal scaffold needs to simulate the three-dimensional structure of extracellular matrix with many advantages: (1) porous structures for supporting cell adhesion, growth, and migration to promote cell scaffold interaction; (2) sufficient elasticity and mechanical properties; (3) controllable degradation rate; (4) uniform distribution of new bone formation to avoid bone necrosis; and (5) minimal inflammation and toxicity in the body (Bose et al., 2012). Based on the rapid development of nanomaterials, the derived composite scaffolds have biological activity and reabsorption to provide good mechanical properties and promote cell adhesion and proliferation. Compared with traditional materials, composite nanomaterials can provide better mechanical properties, maintain bone conductivity and biocompatibility, and promote protein adsorption, cell adhesion, and tissue proliferation and differentiation. Yadav et al. (2019) used molybdenum disulfide nanoflakes (MoS_2_NSs) that reinforced the HAp nanocomposite scaffolds (HAp/MoS_2_NSs) to promote bone regeneration. The cells incubated with HAp@MoS_2_NSs showed higher cell adhesion, cell proliferation, and ALP activity in contrast to HAp. *In vivo* and *in vitro* results of the increased ALP level confirmed that HAp@MoS_2_NSs could promote osteogenic differentiation.

**FIGURE 9:**
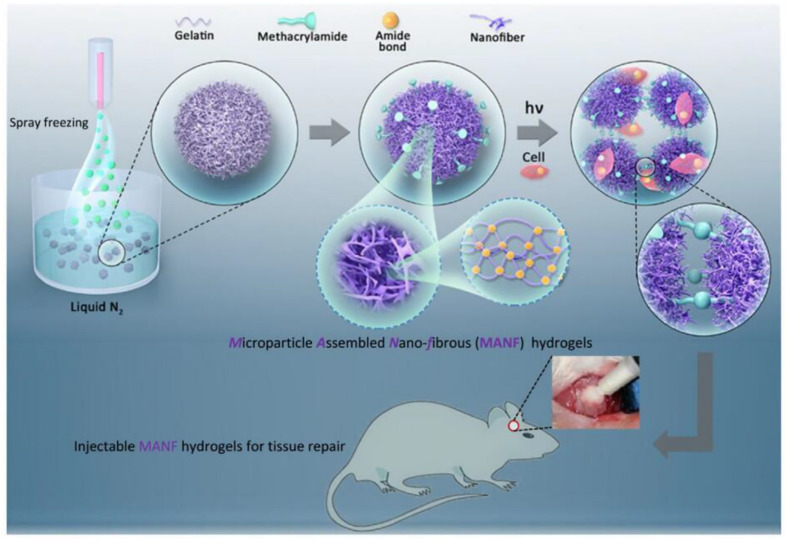
Strategy for fabrication of MANF hydrogels from gelatin nanofibrous microparticles. Reproduced from Hou et al. (2019) with permission from Elsevier (Copyright 2019).

The nanocomposite hydrogel is similar to the extracellular matrix in structure and composition and has a rich interconnected hydrophilic network porous structure, providing greater space for cell attachment and interaction. Hou et al. (2019) reported a new class of injectable hydrogels, microparticle annealed nanofibrous (MANF) hydrogels, which were fabricated via the self-assembly and subsequent crosslinking of gelatin nanofibrous microparticles (NF-MPs). The gelatin solution (ethanol/water mixed solvent) was sprayed into microdroplets from the nozzle and transforms to NF-MPs in the liquid nitrogen bath via the temperature-induced nanoscale phase separation. The gelatin NF-MPs were stabilized via EDC crosslinking and functionalized with the photocrosslinkable methacrylamide groups. The modified NF-MPs could be photo-cured to form an interconnected hydrogel scaffold and cells could be encapsulated during the crosslinking process with a high viability. The hierarchically structured hydrogels supported cell proliferation and osteogenesis *in vitro* and promoted neovascularization and bone defect regeneration *in vivo* (Figure 9).

The nanocomposite fibrous scaffold mimics the fibrous structure of the natural extracellular matrix with a porosity of up to 95%, enhancing the ability of cell adhesion, migration, proliferation, and differentiation in BTE. Yahia et al. (2019) developed a series of electrospun nanofiber scaffolds (NFS) with a sandwich structure based on PCL and chitosan/polyethylene oxide (CS/PEO) composites. On account of the bionic structure, controllable pores, and porous network, these nanocomposite scaffolds could regulate angiogenesis and osteogenesis. The rabbit model experiment of mandibular bone defect *in vivo* also proved that the modified scaffolds could facilitate the fracture healing and bone regeneration.

#### Advances in Tissue-Engineered Bone Technology

Bone bioreactor technology is considered to provide an ideal environment for the combination of seed cells, growth factors, and scaffolds, and control of the bone bioreactor environment has made it possible to prepare isolated tissue-engineered bone (Amini et al., 2012; Tang et al., 2016; Liu et al., 2019). In recent years, a new generation of superimposed manufacturing technology has also received a lot of attention. As a representation, 3D printing manufacturing technology is applied to prepare porous biocompatible scaffolds with excellent mechanical and bone conduction properties (Bittner et al., 2019; Diloksumpan et al., 2020; Polley et al., 2020). Qiao et al. (2020) demonstrated a multifunctional hydrogel scaffold from supramolecular assembly of sodium tetraborate (Na_2_B_4_O_7_), polyvinyl alcohol (PVA), Ag NPs, and tetraethyl orthosilicate (TEOS). These 3D composite scaffolds with suitable pore size and matched bone porosity exhibited good antibacterial and biological activity, which promoted BMSC proliferation and osteogenic differentiation and inhibited bacteria. *In vivo* experiments revealed that the implant showed effective antibacterial ability while promoting bone regeneration as innovative scaffolds for bone regeneration. Sallstrom et al. (2020) prepared a composite material using an extrusion-based additive manufacturing technique with controlled shapes and tunable mechanical properties. These printed structures supported their own weight without requiring curing during printing, which enabled the use of a printing-then-curing approach, by which the cells could grow well on the hydrogel surface in this zwitterionic sulfobetaine hydrogel system. Kankala et al. (2018) demonstrated a 3D porous scaffold using the innovative combinatorial 3D printing and freeze-drying technologies on gelatin (Gel), nano-hydroxyapatite (n-HA), and poly(lactide-co-glycolide) (PLGA) for bone regeneration. These formed Gel/n-HA/PLGA scaffolds possessed good biocompatibility, biodegradation, and mechanical properties, thus promoting cell adhesion, growth, and differentiation with the verification of particular biomarker expression in the ossification process.

Combined with 3D images and CT data analysis, 3D printing scaffolds are precisely prepared with controlled structure, porosity, and property, which can match the specific bone defects. The most extensive and in-depth research of nanomaterial preparation technology includes wet chemical precipitation preparation, sol-gel synthesis technology, hydrothermal synthesis technology, molecular self-assembly technology, and freeze-drying and phase separation, which have significantly promoted the development of nanocomposites (Rezvani et al., 2016; Melo et al., 2019). In addition, genetic engineering has also shown irreplaceable advantages in the application of tissue-engineered bone. Genetic engineering can prolong the expression time of proteins and regulate the expression of transgenes to stimulate bone regeneration and repair (Evans, 2012). Afterward, therapies based on gene expression modification have emerged as a potential alternative therapy for orthopedic diseases. For example, the use of RNA interference-based therapy can effectively target genes that down-regulate bone formation for effective treatment of osteoporosis. Mora-Raimundo et al. (2019) prepared polyethyleneimine-functionalized mesoporous silica nanoparticles (MSNs@PEI). After the combination of the SOST siRNA and the human parathyroid hormone-related peptide, these mesoporous MSNs@PEI nanoparticles could promote the osteoblasts’ growth and differentiation for osteoporosis therapy (Mora-Raimundo et al., 2019).

**FIGURE 10:**
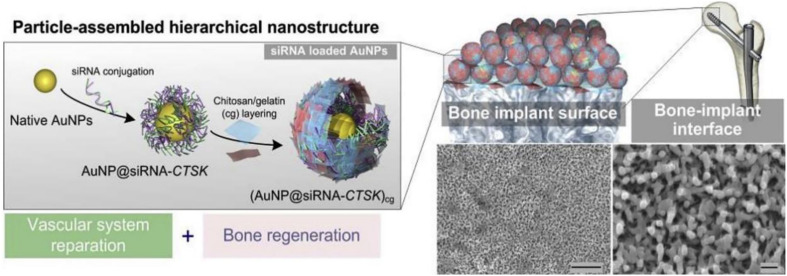
siRNA-decorated nanoparticles were assembled to engineer a hierarchical nanostructured coating on clinically used titanium implants for the synergistic regeneration of skeletal and vascular tissues. Reproduced from Xing et al. (2020) with permission from Elsevier (Copyright 2020).

The weakening ability of bone implant combination has become the main restricting factor for implant treatment in patients with osteoporosis and other patients. The key to solving these problems is to deliver the ideal cell and gene targets efficiently and specifically. Xing et al. (2020) used the LBL self-assembly technology to assemble the Au NPs modified by siRNA-CTSK onto the surface of titanium implant through the bio-based polymer materials and constructed a hierarchical nanostructure coating (Figure 10). The release of siRNA targeted the regulation of cathepsin K and enhanced bone–implant interfacial interaction. siRNA-CTSK could be released and internalized by the adjacent macrophages, demonstrating the synergistic effect on improving osteointegration therapy for *in vitro* and *in vivo* bone regeneration and vascular system repair. Therefore, this coating could slow down siRNA-CTSK to monocytes around the implant, inhibit osteoclastic differentiation, change the cell secretion characteristics, and promote the regeneration of bone and vascular tissue around the titanium implant (Xing et al., 2020). However, highly effective delivery vectors and transfection methods were the focus of current research, and safety was still a main obstacle of gene engineering for bone regeneration (Lu et al., 2013; Park et al., 2019). In view of the positive achievements of gene engineering in BTE, it is expected that new genes or regulatory RNAs will be found and utilized to regulate the expression of proteins and transgenes through gene transfer and regulate the host immune system to inhibit the negative effects on bone healing. The safety of clinical applications and evidence support for evidence-based medicine and other related issues are still the focus of further research for scientists and clinicians in their related fields in the future. Thus, it is believed that development of related technologies could help tissue-engineered bone reach new heights.

## Future Outlook and Conclusion

In summary, there is an urgent need for design concepts and formulation methods to manufacture new bone regeneration and repair scaffolds. Specifically, the field of BTE needs more research to discover the relationship between the composition and material structure on multiple length scales and macroscopic osteogenesis potential. The increasing emphasis on scaffold materials and nanotechnology in the field of BTE has brought huge possibilities for scaffold chemical modification, giving it a revolutionary degree of control. To produce “active” scaffolds specifically manufactured for bone regeneration, an ideal scaffold can be temporarily substituted for natural tissues to interact with the surrounding environment, respond to environmental changes, and actively guide cellular events. These abilities will result in faster bone formation, increased healing time, and rapid recovery of function. An ideal scaffold material should possess the following properties for bone regeneration: (1) basic requirement of excellent biocompatibility to support the adhesion and proliferation of bone-forming cells; (2) high mechanical properties for load-bearing; (3) suitable pore interconnectivity and size for transport of nutrients and oxygen; (4) tailored biodegradation or bioresorbability to provide growth space of new bone tissue; and (5) allowable incorporation of biological cues and signals for cell adhesion, proliferation, metabolism, and differentiation.

Significant progress has been made in the study of various materials and tissue-engineered bones, and different materials have demonstrated fascinating bone regenerative capabilities for the last two decades. With the in-depth study of BTE, researchers have developed various technologies such as electrospinning and molecular self-assembly for successful application into the manufacture of nanofiber scaffolds. Many reports have confirmed that their bionic properties can improve cell adhesion and osteogenic differentiation and organization formation. In essence, scaffold materials will continue to be active participants in the process of bone regeneration as cells and molecular carriers and will play an important role in controlling delivery efficiency and delivery rate. It is worth noting that the degradation of scaffold materials requires the release of biomolecules in a time-dependent manner to prevent the burst of biomolecule release, which is also a basic requirement for new-generation scaffolds. In the future, scholars should devote themselves to studying the interaction between this kind of nano-scaffold material for clinical bone repair and bone regeneration and tissues and to further optimize their composition, structure, and mechanical strength. In addition, the characteristics of different nanocomposites combined with cells also need to be further studied to optimize the survival, adhesion, and migration of the related cells. Thus, we believe that along with clear elucidation of the molecular and signaling mechanisms on tissue repair and regeneration, these series of bone regenerative biomaterials will spark broader interests in the scientific community to create more tailor-made engineering scaffolds with optimum characteristics and advanced properties like natural bones to combat large bone defects in clinical therapeutics.

## Author Contributions

XW, ZT, and XY initiated the project. GT, ZL, YL, and JY searched the database. GT, ZL, and XY wrote, revised, and finalized the manuscript. All authors contributed to the article and approved the submitted version.

## Conflict of Interest

The authors declare that the research was conducted in the absence of any commercial or financial relationships that could be construed as a potential conflict of interest.
